# Diversity, occurrence and conservation of sharks in the southern South China Sea

**DOI:** 10.1371/journal.pone.0213864

**Published:** 2019-03-22

**Authors:** Takaomi Arai, Azie Azri

**Affiliations:** Environmental and Life Sciences Programme, Faculty of Science, Universiti Brunei Darussalam, Jalan Tungku Link, Gadong, BE, Brunei Darussalam; Tanzania Fisheries Research Institute, UNITED REPUBLIC OF TANZANIA

## Abstract

Sharks constitute a vital sector of marine and estuarine nekton and are of great commercial importance all over the world. International concern over the fate of shark fisheries has grown recently. However, information concerning the species diversity, geographic distribution and life histories of sharks in the Indo-Pacific region is highly limited. Comprehensive research on the species composition, distribution and seasonal occurrence of sharks in the southern South China Sea (SSCS) was conducted for four years. A total of 4742 sharks belonging to 10 families and 28 species were recorded from 6 fishing ports in SSCS. The families recorded included Squalidae, Heterodontidae, Orectolobidae, Hemiscylliidae, Alopiidae, Scyliorhinidae, Triakidae, Hemigaleidae, Carcharhinidae and Sphyrnidae. Seventeen of 28 shark species were landed at various developmental stages from in the ranges of or even less than the length at birth and from newborn juveniles to fully-mature. The results suggest that these sharks were born just before fishing and landing, and reproductive-stage sharks were also fished and landed. In total, 15 species, four species and one species in 28 shark species were categorized as Near Threatened, Vulnerable and Endangered species, respectively, on the IUCN Red List. Sharks are not targeted by fisheries practices in the SSCS, but are caught as bycatch throughout the year in various developmental stages. Thus, current fisheries practices in the SSCS area might lead to further decline to critical levels and lead to extinction of some of species in the future. These results suggest that the need for gear selectivity of the commercial fishing gears in order to reduce mortality and to conserve shark stocks.

## Introduction

Sharks are an evolutionarily conservative group, comprising approximately 250 species, ranging in size from 30 cm tiny pygmy shark, *Euprotomicrus bispinatus*, to 12 m plankton-feeding whale shark, *Rhincodon typus*, that have functioned successfully in diverse ecosystems for 400 million years [1]. Sharks constitute an important predator group in marine ecosystems and consequently play an essential role in energy exchange within the highest trophic levels [2]. Despite their evolutionary success, a number of sharks are threatened with extinction as a result of overfishing over recent decades in all oceans [3–5]. The main issues include the high demand for shark fins and gill plates in Asia, unregulated fisheries, bycatch, and increased shark fishing due to the collapse of other fisheries [5, 6–9]. Therefore, sharks are recognized as highly vulnerable to overexploitation leading to population depletion due to their life history strategies [10]. Sharks are predominantly characterized as long-lived and slow growing and they produce few offspring. These characteristics are associated with low productivity, close stock recruitment relationships, and long recovery times in response to overfishing. Other threats such as habitat degradation and environmental contamination through bioaccumulation and biomagnification processes through the food web [11, 12] are serious issues for sharks [13–15].

Shark landings that constitute a part of the demersal fishery occur throughout the southern South China Sea (SSCS) area, especially in Malaysian waters, from the coasts to the edges of its Exclusive Economic Zone [16]. Fisheries in Malaysia and other Southeast Asian countries are expanding rapidly, new fisheries are being actively developed, and the trade in and value of shark products are increasing [17]. In many Southeast Asian countries, drastic increases in fishing effort and shark landings have been followed by marked declines in shark catch rates in fisheries, and a fall in the numbers and biodiversity of sharks entering markets from coastal waters has been detected [17]. Some historically common species no longer appear to be present in some areas. Multispecies fisheries could potentially result in the local extinction of rare sharks taken as bycatch and even the complete extinction of rare regional endemics [17]. The lack of management of shark fisheries is therefore cause for concern. Sharks are not targeted by fishers but are caught together with other commercially important species in SSCS areas [16].

In Southeast Asia, scientific information on sharks is sporadically documented. A large knowledge gap exists regarding the shark population trends within Southeast Asia, the major global consumers of sharks [18]. A major obstacle to the conservation and management of shark populations in the world is the lack of information on the diversity, seasonal occurrence, life history and fisheries of sharks in many areas. A few studies and publications addressing the diversity, species composition and life history of shark are available for the SSCS areas. Of the 109 species present historically in the South China Sea (SCS) [18], 48, 63 and 53 shark species have been reported in 1999 [19], 2004 [20] and 2005 [21], respectively in SSCS areas. This information suggests that approximately half of the SCS’s shark species occur in Malaysian waters. Although few fisheries reports and taxonomical information of sharks from SSCS waters are available [16, 19–21], biological and ecological studies are at a rudimentary level. Therefore, studies on the diversity, seasonal occurrence and landing patterns of sharks in SSCS are urgently needed to provide information not only for fundamental biology and ecology of sharks but also for conservation and fisheries management and assessment of sharks.

In the present study, the species diversity and seasonal occurrence of sharks has been comprehensively researched at six landing ports in SSCS areas of the eastern coast of the Peninsular Malaysia (West Malaysia) and the western coast of Borneo Island (East Malaysia) over four years. We also discuss the current status of the impacts of fisheries on sharks in SSCS areas. The results are relevant for shark conservation and protection of shark diversity.

## Results

**Fig 1:**
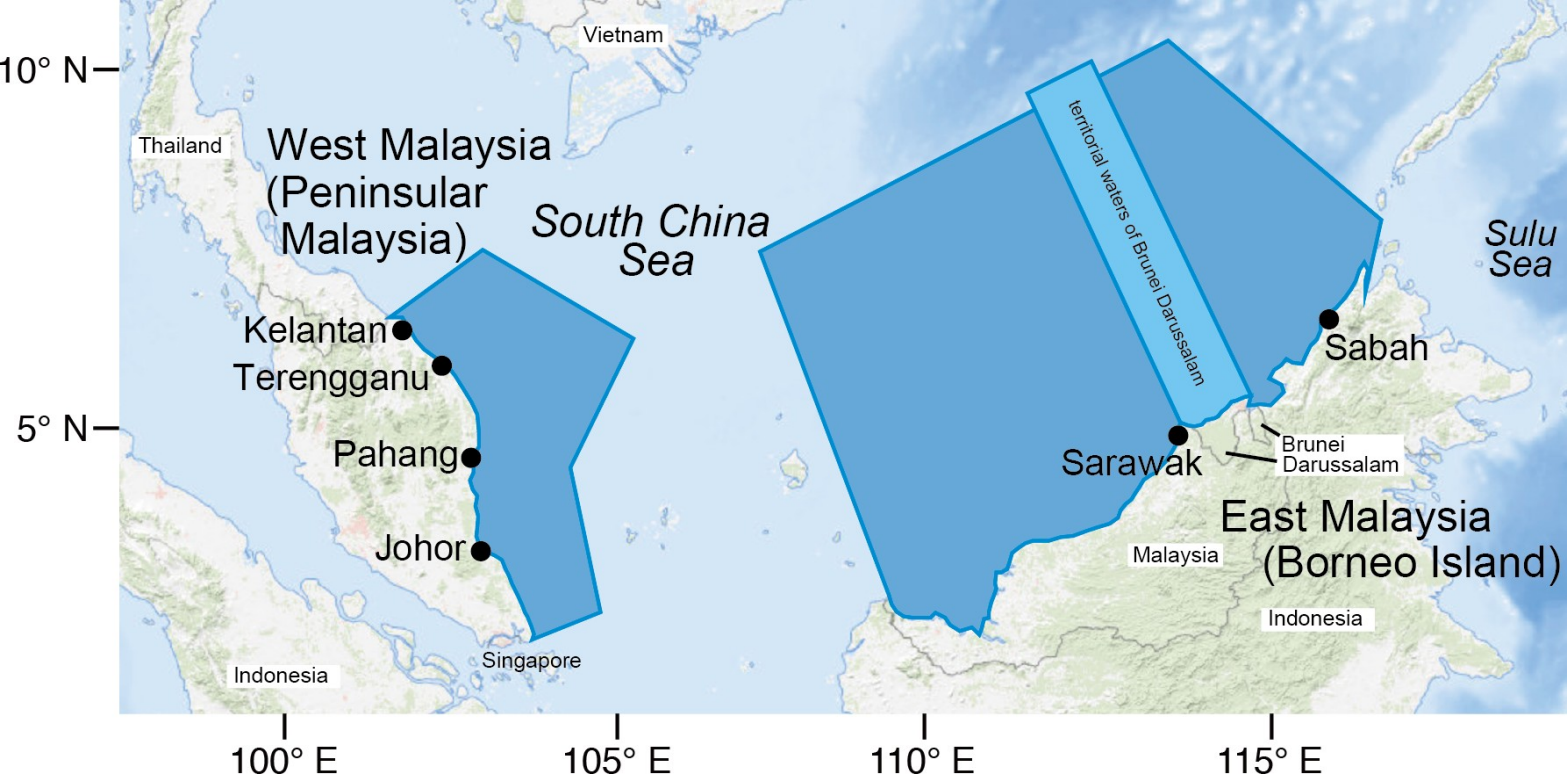
Map of sampling locations for sharks on the eastern coast of the Peninsular Malaysia (West Malaysia) and the western coast of Borneo Island (East Malaysia). All study sites face to the SSCS in Malaysia territorial waters (blue shades) covering the four states of Kelantan, Terengganu, Pahang and Johor in West Malaysia and the two states, Sabah and Sarawak, in East Malaysia. Base map is downloaded from the USGS National Map Viewer (open access) at http://viewer.nationalmap.gov/viewer/.

A total of 4742 shark specimens composed of 28 species from ten families—Squalidae, Heterodontidae, Orectolobidae, Hemiscylliidae, Alopiidae, Scyliorhinidae, Triakidae, Hemigaleidae, Carcharhinidae and Sphyrnidae—were found in the SSCS (Fig 1, Table 1). Carcharhinidae dominated with 14 species, followed by Hemiscylliidae with 5 species, and the other families were represented by one to two species (Table 2). Among the 28 species found in this study, the brownbanded bamboo shark *Chiloscyllium punctatum*, had the highest number of landings with a total of 1355 specimens (29%). The second most dominant species was the Indonesian bamboo shark *Chiloscyllium hasseltii* (871 specimens, 18%), followed by the spot-tail shark *Carcharhinus sorrah* (604 specimens, 13%), the whitespotted bamboo shark *Chiloscyllium plagiosum* (367 specimens, 8%), and the sliteye shark *Loxodon macrorhinus*, which registered 364 specimens (8%) (Table 2). The scalloped hammerhead shark, *Sphyrna lewini*, which is listed as an Endangered species on the IUCN Red List [22] (Table 1), was also abundant (184 specimens, 4%). Furthermore, *Alopias pelagicus*, *Hemigaleus microstoma*, *Hemipristis elongata* and *Carcharhinus plumbeus* are listed as Vulnerable species, while 15 of the 28 shark species are listed as Near Threatened on the IUCN Red List [22] (Table 1).

**Table 1:** Sharks landed in the southern South China Sea and their depth preference, habitat and distribution [24, 43–44, 46–59] and IUCN status [22].

Species	Depth	Habitat	Distribution	Reference	IUCN status
	preference (m)				
*Squalus altipinnis*	50 to 732	continental shelves and upper slopes	temperate and tropical seas	44	Data Deficient
		on or near the bottom	Eastern Atlantic, western Indian Ocean,		
			western Pacific and Australia		
*Heterodontus zebra*	50 to 200	bottom of continental	Western Pacific,	46,47,48	Least Concern
		and insular shelves	northern western Australia		
*Orectolobus leptolineatus*	20 to at	inshore to offshore bottom of continental shelves,	tropical and warm temperate waters,	44	Not Evaluated
	least 110	juveniles occurs in low reefs, seagrass beds	Western Pacific		
		and estuaries			
*Chiloscyllium hasseltii*	0 to 12	inshore bottom dweller, rock and coral reefs,	Indo-west Pacific, Eastern Indian Ocean	46, 49, 50	Near Threatened
		sandy and muddy bottom	and western central Pacific		
*Chiloscyllium indicum*	0 to 90	demersal inshore, possibly enter brackish water	Indo-west Pacific region	47, 51, 52	Near Threatened
		and freshwater			
*Chiloscyllium plagiosum*	0 to 50	inshore reef dwelling,	Indo-west Pacific region	46, 50, 51	Near Threatened
		rocks and coral reefs			
*Chiloscyllium punctatum*	limit to 85	nearshore intertidal and subtidal habitat, coral reef,	Indo-west Pacific region,	53	Near Threatened
		seagrass beds and rocky, sandy and muddy substrates	tropical and warm-temperate waters		
*Alopias pelagicus*	surface to	oceanic and nearshore	Oceanic and wide-ranging	44, 46	Vulnerable
	at least 152	highly migratory and is epipelagic	subtropical and tropical Indo-Pacific		
*Atelomycterus marmoratus*	limit to 15	inshore coral reefs	tropical region of	24, 47	Near Threatened
		inhabit crevices and holes on reefs	Indo-west Pacific		
*Halaelurus buergeri*	80 to 100	continental shelf, bottom dwelling	western north Pacific	45, 46, 54	Data Deficient
*Mustelus widodoi*	20 to 250	demersal on mid continental shelf to upper slopes	Indian Ocean	45, 46, 54	Data Deficient
		in deep water, inshore and offshore and sometimes			
		on coral reef			
*Hemigaleus microstoma*	surface to	continental and insular shelves	Indo-west Pacific region	44, 55	Vulnerable
	at least 170	in shallow waters			
*Hemipristis elongata*	1 to 130	continental and insular shelves	Indo-west Pacific region	53, 54, 56	Vulnerable
	
*Carcharhinus amblyrhynchoides*	10 to 50	coastal pelagic on continental and insular shelves	tropical Indo-West Pacific	46, 56, 57	Near Threatened

*Carcharhinus brevipinna*	surface to	continental and insular shelves from close inshore	warm temperate, subtropical	43, 47	Near Threatened
	at least 75	to offshore,nearshore waters off beaches, in bays	and tropical Atlantic, Indian		
		and off river mouths, pelagically offshore	and Westerns Pacific Oceans		
*Carcharhinus tjutjot*	surface to 170	demersal inshore	tropical Indo-West Pacific	43, 57	Near Threatened

*Carcharhinus leucas*	1 to 150	coastal, estuarine and freshwater	tropical and warm	43, 47, 57	Near Threatened
			temperate areas		
*Carcharhinus limbatus*	0 to 30	inshore and offshore, pelagic over continental	widespread in warm temperate,	43, 47, 58	Near Threatened
		and insular shelves, off river mouthsand estuaries,	subtropical and tropical waters		
		muddy bays, mangrove swamps, lagoons,			
		and coral reef drop-offs			
*Carcharhinus melanopterus*	20 to 75	insular shelves of shallow reefs	tropical Indo-West Pacific	47, 58, 59	Near Threatened
		and occasionally present in brackish water	and Central Pacific		
*Carcharhinus plumbeus*	surface to	inshore and offshore, continental and insular shelves	world-wide tropical and	43, 57	Vulnerable
	at least 280		warm temperate waters		
*Carcharhinus sealei*	surface to 40	coastal, continental and insular shelves,	Indo-west Pacific region	47,57	Near Threatened
		from the surf line and intertidal region to deeper water			
*Carcharhinus sorrah*	20 to 140	continental and insular shelves, mud and sand bottom,	tropical Indo-West Pacific	47, 56, 57	Near Threatened
		near coral reefs, shallow water, water column			
		but mainly in midwater or near the surface			
*Galeocerdo cuvier*	0 to 150	continental and insular shelves, inshore, river estuaries,	worldwide tropical and	43, 47	Near Threatened
		harbors, coral atolls and lagoons	warm temperate seas		
*Loxodon macrorhinus*	7 to 100	continental and insular shelves	Indo-west Pacific region	43, 57	Least Concern
	
*Rhizoprionodon acutus*	1 to 200	continental shelves, often on sandy	tropical areas of eastern Atlantic,	43, 47	Least Concern
		beach, rarely estuaries, mid water	Indo-west Pacific		
*Scoliodon macrorhynchos*	no information	shallow water, insular shelves close inshore,	tropical Indo-West Pacific	43	Not Evaluated
		rocky areas			
*Triaenodon obesus*	1 to 40	continental shelves, coral reefs,	Indo-Pacific oceans	43, 47, 57	Near Threatened
		and island terraces			
*Sphyrna lewini*	surface to	continental and insular shelves	warm temperate and tropical seas	43, 47	Endangered
	at least 275	coastal and semi-oceanic pelagic			

**Table 2:** Number of sharks and species composition landed in the southern South China Sea.

Order	Family/Species	Common name	Total landing	Composition
			(number)	(%)
Squaliformes	Squalidae			
	*Squalus altipinnis*	Piked spurdog	39	1
Heterodontiformes	Heterodontidae			
	*Heterodontus zebra*	Zebra horn shark	3	0.06
Orectolobiformes	Orectolobidae			
	*Orectolobus leptolineatus*	Indonesian wobbegong	1	0.02
	Hemiscylliidae			
	*Chiloscyllium hasseltii*	Indonesian bamboo shark	871	18
	*Chiloscyllium indicum*	Slender bamboo shark	10	0.21
	*Chiloscyllium plagiosum*	Whitespotted bamboo shark	367	8
	*Chiloscyllium punctatum*	Brownbanded bamboo shark	1355	29
Lamniformes	Alopiidae			
	*Alopias pelagicus*	Pelagic thresher	5	0.11
Carcharhiniformes	Scyliorhinidae			
	*Atelomycterus marmoratus*	Coral catshark	122	2
	*Halaelurus buergeri*	Blackspotted catshark	3	0.06
	Triakidae			
	*Mustelus widodoi*	Arabian smooth-hound	3	0.06
	Hemigaleidae			
	*Hemigaleus microstoma*	Weasel shark	119	2
	*Hemipristis elongata*	Fossil shark	5	0.11
	Carcharhinidae			
	*Carcharhinus amblyrhynchoides*	Graceful shark	7	0.15
	*Carcharhinus brevipinna*	Spinner shark	121	2
	*Carcharhinus tjutjot*	Whitecheek shark	12	0.25
	*Carcharhinus leucas*	Bull shark	7	0.15
	*Carcharhinus limbatus*	Blacktip shark	12	0.25
	*Carcharhinus melanopterus*	Blacktip reef shark	5	0.11
	*Carcharhinus plumbeus*	Sandbar shark	4	0.08
	*Carcharhinus sealei*	Blackspot shark	185	4
	*Carcharhinus sorrah*	Spot-tail shark	604	13
	*Galeocerdo cuvier*	Tiger shark	7	0.15
	*Loxodon macrorhinus*	Sliteye shark	364	8
	*Rhizoprionodon acutus*	Milk shark	287	6
	*Scoliodon macrorhynchos*	Pacific spadenose shark	39	1
	*Triaenodon obesus*	Whitetip reef shark	1	0.02
	Sphyrnidae			
	*Sphyrna lewini*	Scalloped hammerhead shark	184	4

**Fig 2:**
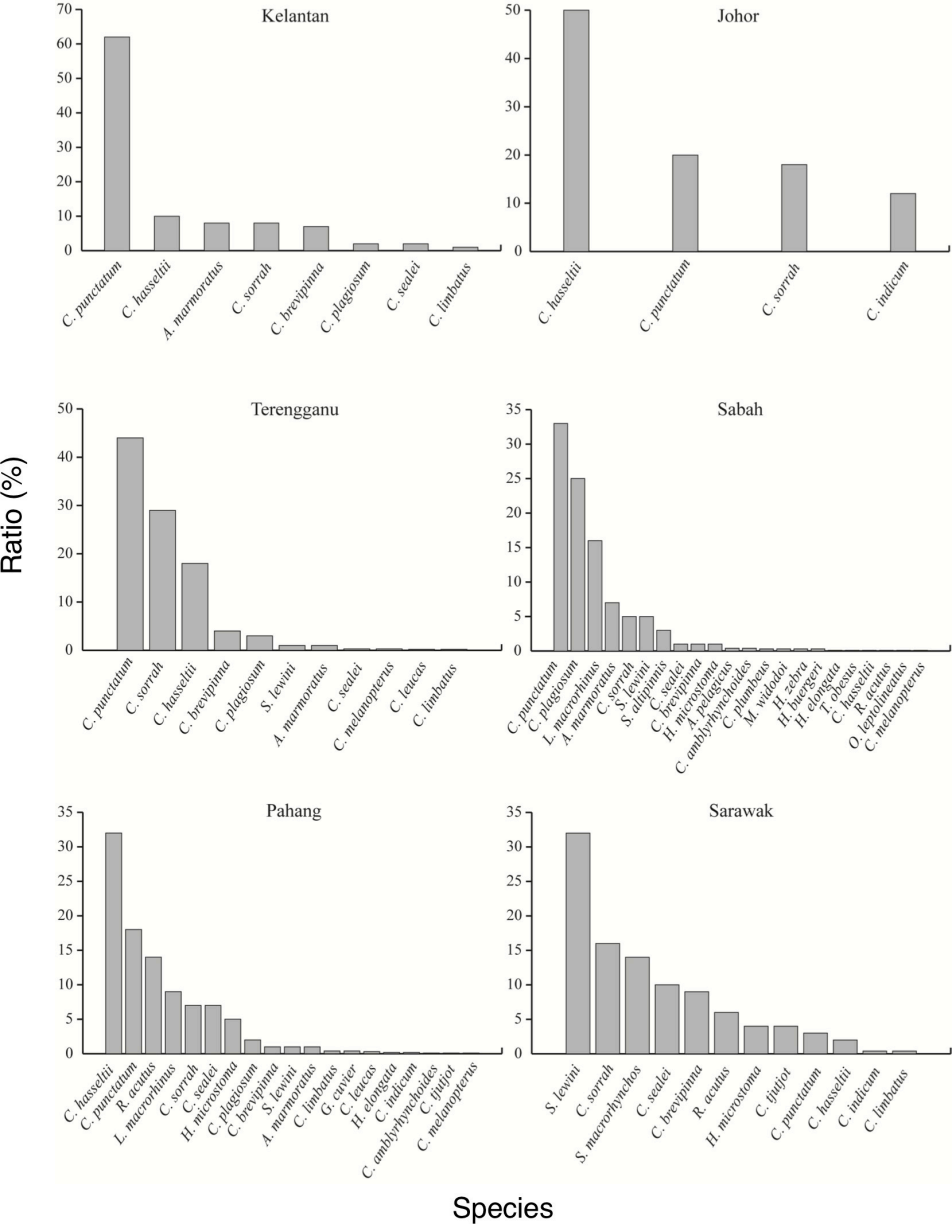
Shark diversity landed in six states in the southern South China Sea.

Seventeen of the 28 species commonly occurred in both West and East Malaysia, while 2 species (*Carcharhinus leucas* and *Galeocerdo cuvier*) occurred in West Malaysia and 9 species (*Squalus altipinnis*, *Heterodontus zebra*, *Orectolobus leptolineatus*, *Alopias pelagicus*, *Halaelurus buergeri*, *Mustelus widodoi*, *Carcharhinus plumbeus*, *Scoliodon macrorhynchos* and *Triaenodon obesus*) occurred in East Malaysia (Fig 2, Table 3). The brownbanded bamboo shark *Chiloscyllium punctatum* or the Indonesian bamboo shark *Chiloscyllium hasseltii* were the most dominant species in all sites except Sarawak State (Fig 2). The scalloped hammerhead shark *Sphyrna lewini* was the most dominant species in Sarawak state (Fig 2). Although we conducted much more intensive landing port surveys in West Malaysia (4 sites and 43 times (months)) than in East Malaysia (2 sites and 7 times (months)), more shark species were observed in East Malaysia (26 species) than in West Malaysia (19 species) (Table 3). The results suggest that shark diversity is higher in the eastern areas than the western areas of the SSCS.

**Table 3:** Sharks landed on the eastern coast of the Peninsular Malaysia (West Malaysia) and the western coast of Borneo Island (East Malaysia) and their overlapping species.

Peninsular Malaysia	Borneo Island	Peninsular Malaysia and
(West Malaysia)	(East Malaysia)	Borneo Island
*Carcharhinus leucas*	*Squalus altipinnis*	*Chiloscyllium hasseltii*
*Galeocerdo cuvier*	*Heterodontus zebra*	*Chiloscyllium indicum*
	*Orectolobus leptolineatus*	*Chiloscyllium plagiosum*
	*Alopias pelagicus*	*Chiloscyllium punctatum*
	*Halaelurus buergeri*	*Atelomycterus marmoratus*
	*Mustelus widodoi*	*Hemigaleus microstoma*
	*Carcharhinus plumbeus*	*Hemipristis elongata*
	*Scoliodon macrorhynchos*	*Carcharhinus amblyrhynchoides*
	*Triaenodon obesus*	*Carcharhinus brevipinna*
		*Carcharhinus tjutjot*
		*Carcharhinus limbatus*
		*Carcharhinus melanopterus*
		*Carcharhinus sealei*
		*Carcharhinus sorrah*
		*Loxodon macrorhinus*
		*Rhizoprionodon acutus*
		*Sphyrna lewini*

**Fig 3:**
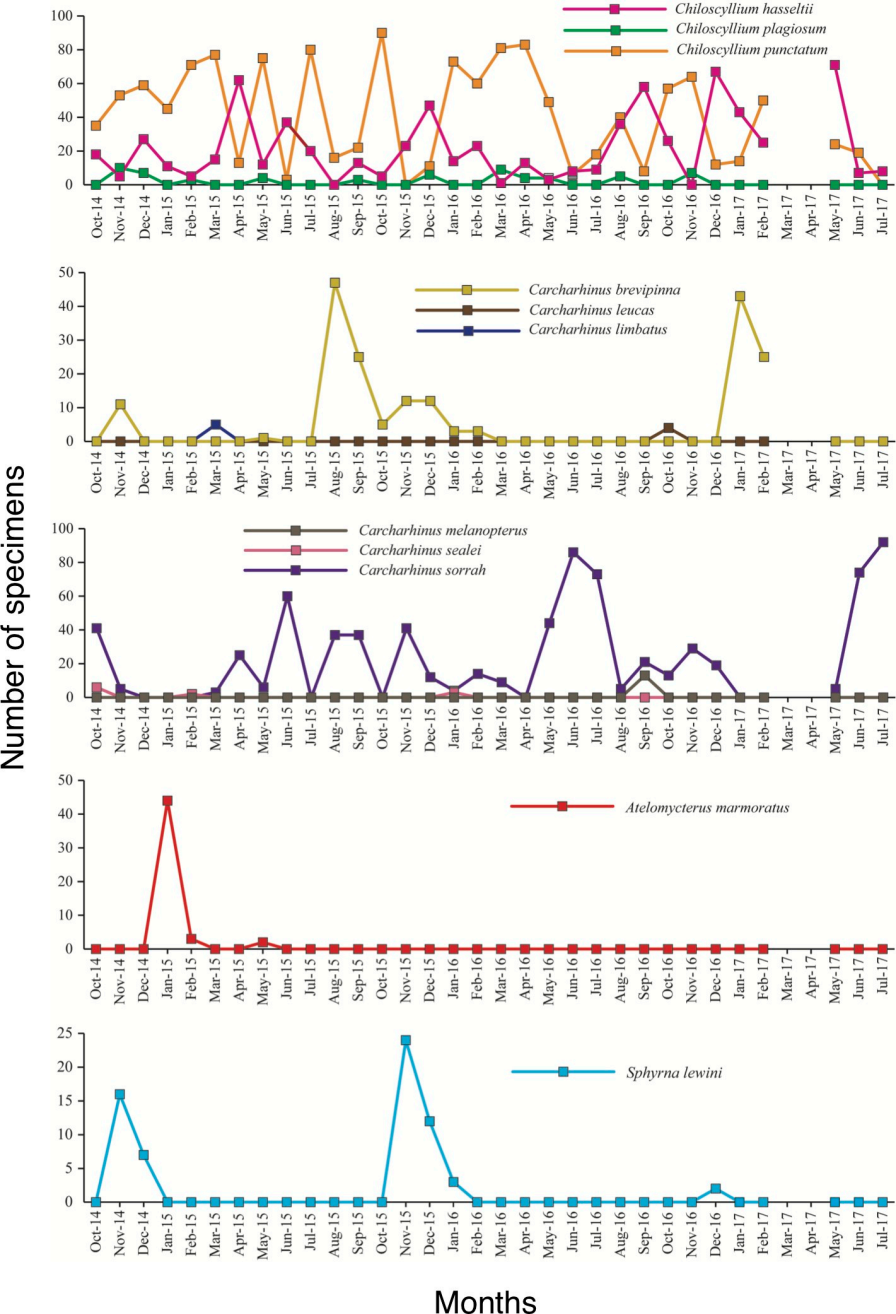
Seasonal occurrence and diversity of sharks landed in Terengganu State in the southern South China Sea. Eleven species were found in monthly observations for approximately four years from 2014 to 2017. Three species, the brownbanded bamboo shark *Chiloscyllium punctatum*, the spot-tail shark *Carcharhinus sorrah* and the Indonesian bamboo shark *Chiloscyllium hasseltii*, were the dominant species constituting 90% of sharks of a total of 11 shark species in the Terengganu State.

In Terengganu State, 11 species were found in monthly observations for approximately four years (Fig 3). Three species, the brownbanded bamboo shark *Chiloscyllium punctatum*, the spot-tail shark *Carcharhinus sorrah* and the Indonesian bamboo shark *Chiloscyllium hasseltii*, were the most dominant species comprising 90% of the shark species in the Terengganu State (Fig 3). The seasonal occurrences in the three dominant species of *C*. *punctatum*, *C*. *hasseltii* and *C*. *sorrah* were similar among the years (Fig 3). Eight of the 11 shark species were sporadically found among months and years (Fig 3). There were significant monthly fluctuations in the abundance of *C*. *sorrah* (one-way ANOVA, F_11, 31_ = 3.198, p < 0.05). However, *C*. *punctatum* and *C*. *hasseltii* did not show significant monthly fluctuations (one-way ANOVA, F_11, 31_ = 1.701–1.984, p > 0.05). *C*. *punctatum* dominated throughout the years with the highest abundances from January to May and October and November (Fig 3). *C*. *sorrah* and *C*. *hasseltii* were the second and third dominant species, respectively, occurring throughout the years (Fig 3). *C*. *sorrah* was the most abundant in June and July for three years between 2015 and 2017 (Fig 3). *C*. *punctatum* and *C*. *hasseltii* were in the adult stages without newborn juvenile specimens throughout the years. The smallest sizes of *C*. *punctatum* and *C*. *hasseltii* were 24.0 cm and 38.1 cm, respectively, in male for both species.

**Fig 4:**
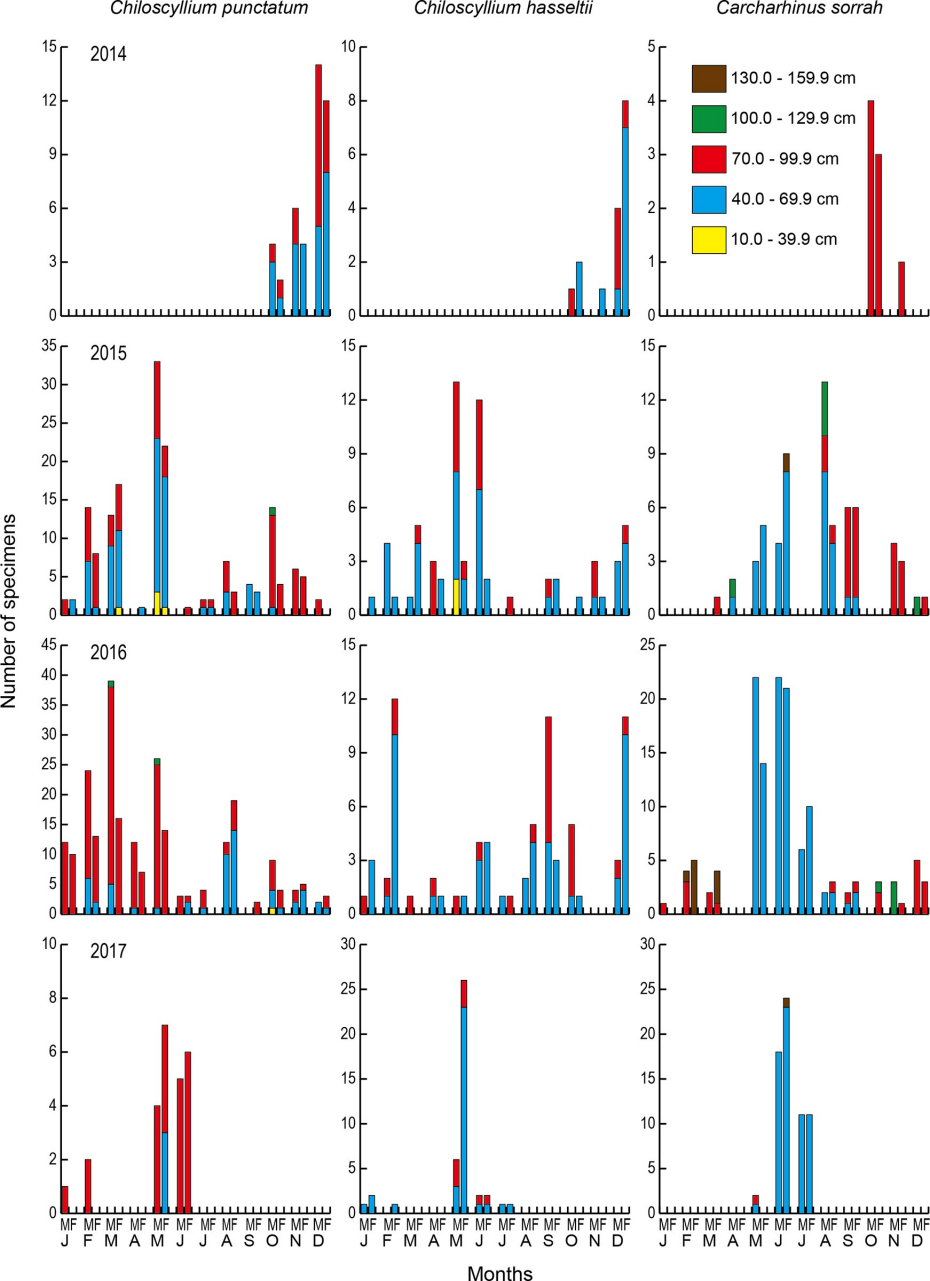
Monthly size distributions of three dominant species, the brownbanded bamboo shark *Chiloscyllium punctatum*, the Indonesian bamboo shark *Chiloscyllium hasseltii* and the spot-tail shark *Carcharhinus sorrah*, landed in Terengganu State in the southern South China Sea. Monthly size distributions were recorded for approximately four years from 2014 to 2017.

The TL distribution of the three most dominant species (*C*. *punctatum*, *C*. *hasseltii* and *C*. *sorrah*) in Terengganu State were significantly different among months and years (Kruskal-Wallis test, H_3_ ≤ 2.712, p < 0.005). For *C*. *sorrah*, most of the specimens found in June and July were in the newborn stage, that is less than 60 cm, while the sharks in the other months were mostly at the adult stage (> 70 cm) (Fig 4). The total lengths in June and July in 2015 and 2016 were significantly smaller than other months in both sexes (Mann-Whitney-*U* test, 16 ≤ U ≤ 706.5, 30 ≤ df ≤ 43, p < 0.005), while no significant difference was found between June and July and other months in 2015 for female (Mann-Whitney-*U* test, U = 141, df = 8, p > 0.05). Significant differences in TL were found between four years in each sex in *C*. *punctatum* (Kruskal-Wallis test, H_3_ ≤ 2.652, p < 0.005) with the exception between 2014 and 2015 for males and between 2014 and 2015 and between 2016 and 2017 for females (Mann-Whitney-*U* test, 631.5 ≤ U ≤ 1193, 16 ≤ df ≤ 43, df = 16–43, p > 0.05). However, there were no significant differences in TL between four years in both sexes in *C*. *hasseltii* (Kruskal-Wallis test, H_3_ ≤ 2.712, p > 0.05) except for males during 2014 and 2015 (Mann-Whitney-*U* test, U = 148, df = 10, p < 0.05). In *C*. *sorrah*, four out of six TL combinations were significantly different between years for males (Mann-Whitney-*U* test, 36 ≤ U ≤ 646.5, 7 ≤ df ≤ 79, p < 0.05) and significant differences were found in three out of six TL combinations for females (Mann-Whitney-*U* test, 4 ≤ U ≤ 919.5, 28 ≤ df ≤ 99, p < 0.05).

**Fig 5:**
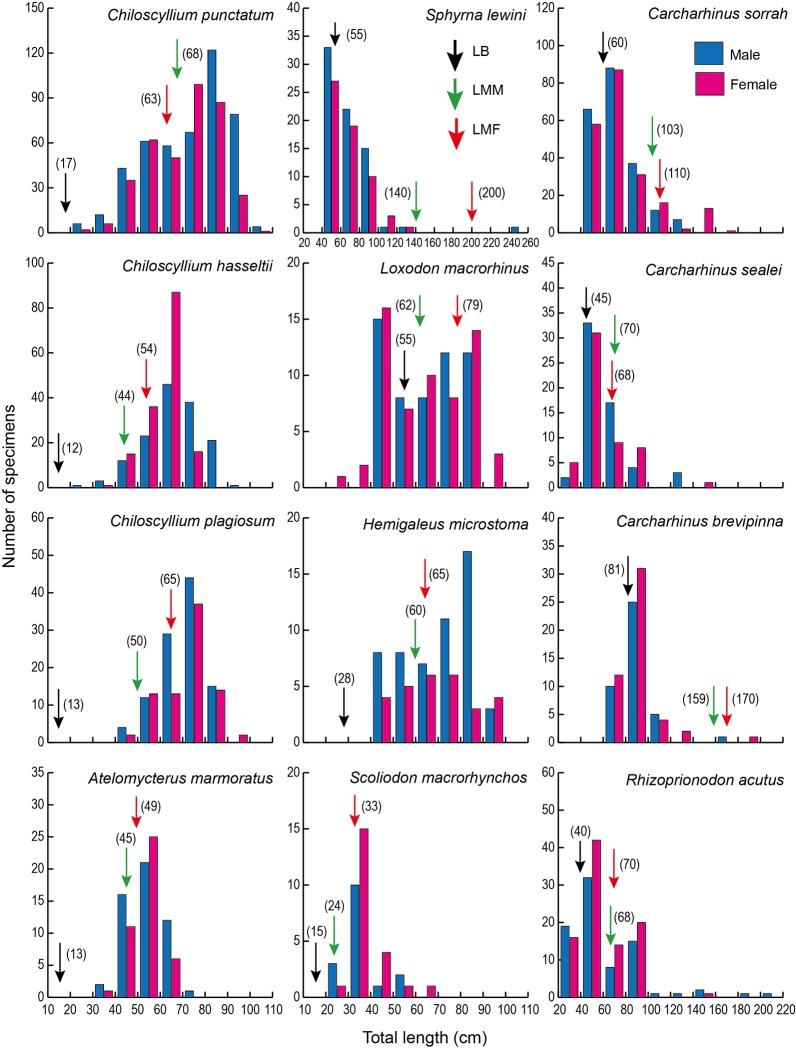
Size distributions of sharks landed in the southern South China Sea. Twelve shark species with more than 20 specimens landed at various growth stages from newborn juveniles and/or to mature adults^22-26^. LB (black arrow), LMM (green arrow) and LMF (red arrow) indicate the length at birth, length at maturation of male and length at maturation of female, respectively.

**Fig 6:**
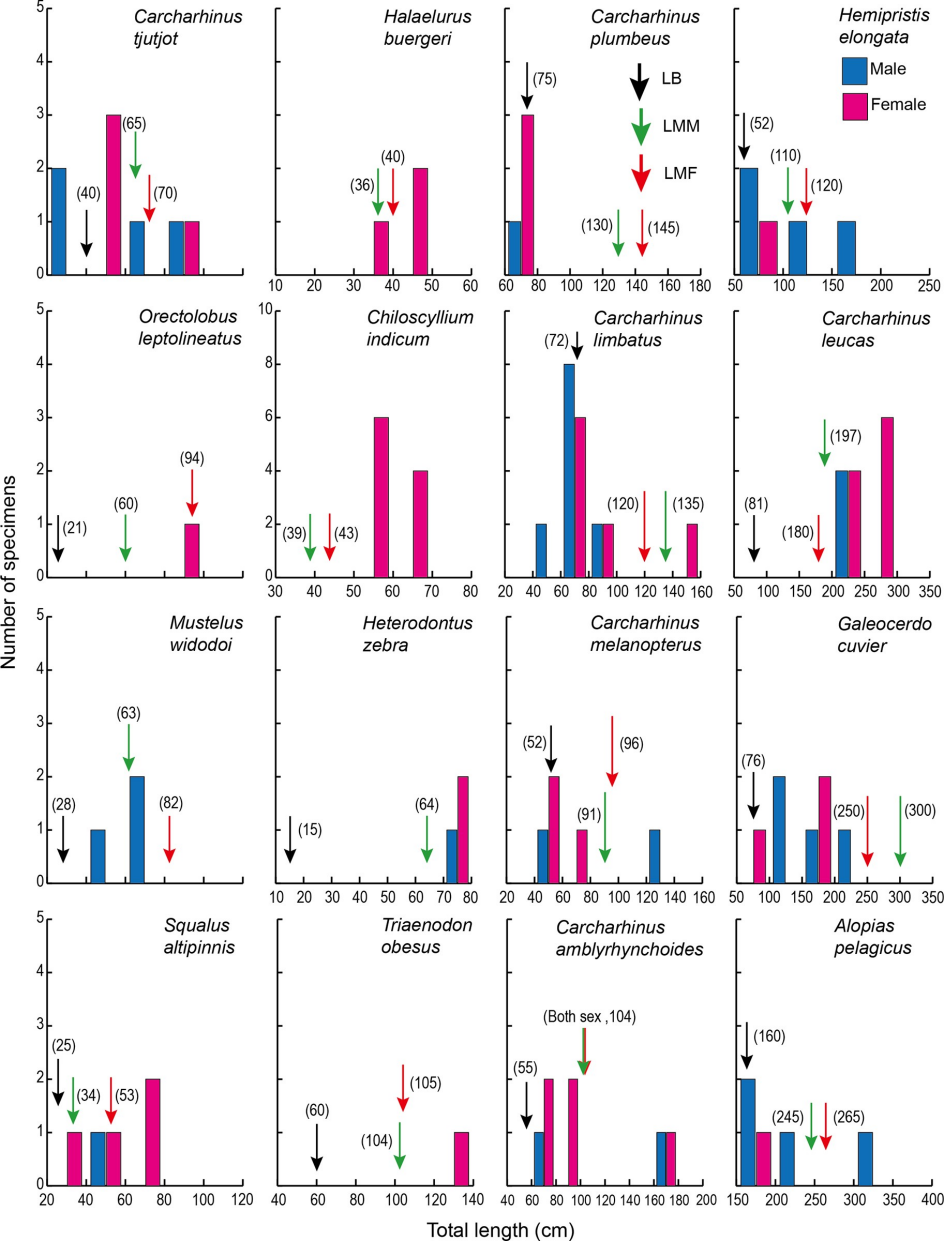
Size distributions of sharks landed in the southern South China Sea. Sixteen shark species with less than 5 specimens were landed at various growth stages from the newborn juveniles and/or to mature adults^22-26^. LB (black arrow), LMM (green arrow) and LMF (red arrow) indicate the length at birth, length at maturation of male and length at maturation of female, respectively.

**Fig 7:**
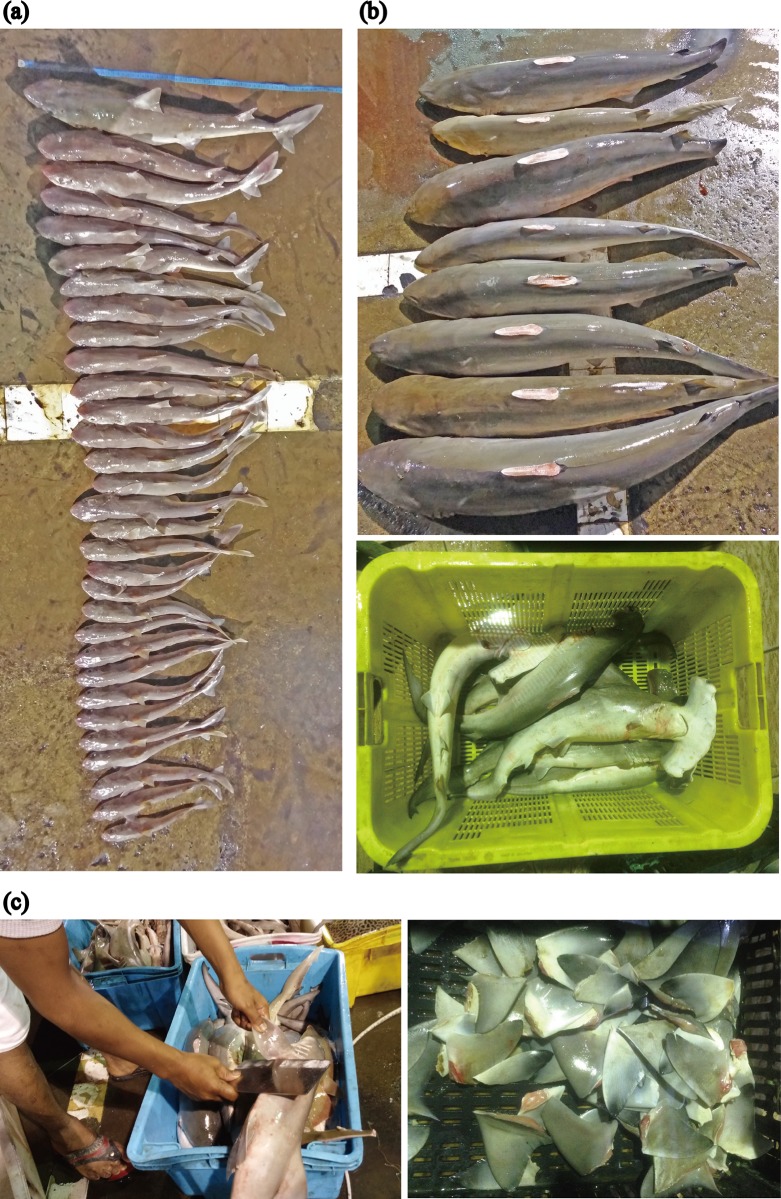
Sharks landed in the southern South China Sea. Piked spurdog *Squalus altipinnis* at various growth stages from the newborn to mature adults were landed (a). Sharks with fins cut off at landing ports in southern South China Sea (b). Shark finning practice at landing ports in southern South China Sea (c).

The size ranges varied among species, ranging from 20 to 309 cm TL (Figs 5 and 6) and from 0.03 to 26.9 kg body weight (Table 4). The TL at birth varied among species [22–26] (Table 4). Various growth stages from newborn to the mature adults were landed (Figs 5 and 6). Ten of the 28 captured shark species, *Squalus altipinnis*, *Carcharhinus brevipinna*, *C*. *tjutjot*, *C*. *limbatus*, *C*. *plumbeus*, *C*. *sealei*, *C sorrah*, *Loxodon macrorhinus*, *Rhizoprionodon acutus* and *Sphyrna lewini* were in or less than the length at birth (Figs 5, 6 and 7A, Table 4). Although the TL of seven of the shark species, *Chiloscyllium hasseltii*, *C*. *punctatum*, *Atelomycterus marmoratus*, *Carcharhinus amblyrhynchoides*, *C*. *melanopterus*, *Hemigaleus microstoma* and *Scoliodon macrorhynchos*, were greater than the TL at birth, various growth stages from newborn juveniles to mature adults were landed (Figs 5 and 6).

**Table 4:** Total lengthand body weight of sharks in this study and total length at birth, hatch and maturity of sharks [22–26].

Species	Sex	Number of spcimens examined	Total length (cm)		Number of spcimens examined	Body weight (kg)		Total length at	Total length at
			Range	Mean ± SD		Range	Mean ± SD	birth/hatch (cm)	maturity (cm)
*Squalus altipinnis*	Female	1	54.5		1	0.37		20–25	34–41
	Male	4	39–73	59.9 ± 15.3	4	0.25–1.75	1.0 ± 0.7		53–57
*Heterodontus zebra*	Female	1	73.5		1	1.95		15	64
	Male	2	71–78.5	74.8 ± 5.3	2	2.59–4.63	3.6 ± 1.4		no data
*Orectolobus leptolineatus*	Female	0						21	60
	Male	1	95		1	6.85			94
*Chiloscyllium hasseltii*	Male	145	27.5–90.5	66.5 ± 12.2	145	0.18–3.4	1.4 ± 0.7	9–12	44–54
	Female	155	37.5–77	61.7 ± 7.9	155	0.2–2.44	1.1 ± 0.4		54–59
*Chiloscyllium indicum*	Male	0						no data	39–42
	Female	10	54–63	58.6 ± 3.0	10	0.42–0.76	0.6 ± 0.1		43
*Chiloscyllium plagiosum*	Male	104	41–89	70.2 ± 9.9	104	0.18–3.6	1.0 ± 0.5	10–13	50–63
	Female	81	46–93	71.3 ± 10.6	81	0.34–2.12	1.2 ± 0.5		65
*Chiloscyllium punctatum*	Male	452	20.7–105	72.7 ± 17.9	452	0.03–4.6	1.7 ± 1.1	13–17	68–76
	Female	367	20–101	70.2 ± 15.2	367	0.08–4.04	1.5 ± 0.9		63
*Alopias pelagicus*	Female	4	182–308.5	226.1 ± 58.3	3	11.4–25	16.7 ± 7.3	130–160	245–270
	Male	1	178		1	11			265–290
*Atelomycterus marmoratus*	Male	52	31–70	53.2 ± 7.6	52	0.08 -.1.12	0.5 ± 0.2	10–13	45–47
	Female	43	37–68	53.0 ± 6.3	43	0.15 -. 0.95	0.5 ± 0.2		49
*Halaelurus buergeri*	Female	0						no data	36–43
	Male	3	39.5–44.4	42.3 ± 2.5	3	0.19–0.31	0.3 ± 0.1		40
*Mustelus widodoi*	Female	3	55–67	61.3 ± 6.0	3	0.47–0.89	0.7 ± 0.2	26–28	63–67
	Male	0							82
*Hemigaleus microstoma*	Female	52	40.4–99	69.3 ± 15.3	52	0.22–2.84	1.4 ± 0.8	26–28	60
	Male	27	41.5–95	68.4 ± 16.3	27	0.21–4.18	1.4 ± 1.1		65
*Hemipristis elongata*	Female	4	62.5–155	101.3 ± 43.1	3	0.77–19	7.1 ± 10.3	45–52	110
	Male	1	69		1	1.18			120
*Carcharhinus amblyrhynchoides*	Male	2	62.5–169	115.8 ± 75.3	1	1.77		52–55	104–115
	Female	5	61–162	96.3 ± 39.1	4	1.62–5.88	3.9 ± 2.1		104–115
*Carcharhinus brevipinna*	Male	41	69–165	87.4 ± 15.4	40	1.3–7.15	3.3 ± 1.4	60–81	159–203
	Female	50	69.5–180	89.2 ± 18.4	48	1.9–16.38	3.5 ± 2.3		170–220
*Carcharhinus tjutjot*	Male	4	38.4–86.0	58.1 ± 23.7	4	0.28–3.68	1.5 ± 1.6	28–40	65–75
	Female	4	40.5–88.5	56.9 ± 21.5	2	0.46–4.18	2.3 ± 2.6		70–75
*Carcharhinus leucas*	Male	2	200–225	212.5 ± 17.7	0			55–81	197–226
	Female	5	210–278.5	248.7 ± 34.0	0				180–230
*Carcharhinus limbatus*	Male	6	57–82.5	72.7 ± 9.4	6	1.1–3.1	2.1 ± 0.8	38–72	135–180
	Female	5	65.5–150	87.9 ± 35.1	4	1.85–2.94	2.3 ± 0.5		120–190
*Carcharhinus melanopterus*	Male	2	55.5–122	88.8 ± 47.0	2	0.93–12.3	6.6 ± 8.0	33–52	91–113
	Female	3	56–61.3	57.9 ± 3.0	3	0.76–1.18	0.9 ± 0.2		96–120
*Carcharhinus plumbeus*	Male	1	65.5		1	1.42		52–75	130–180
	Female	3	66.0–70.0	68.0 ± 2.0	3	1.39–1.9	1.6 ± 0.3		145–185
*Carcharhinus sealei*	Male	59	37–132.5	60.3 ± 21.1	57	0.21–11.82	1.3 ± 1.6	33–45	70–80
	Female	54	29.5–148.5	57.7 ± 20.3	52	0.12–3.74	1.2 ± 1.1		68–75
*Carcharhinus sorrah*	Male	210	47.5–131.3	71.8 ± 17.8	207	0.32–12.52	2.2 ± 2.1	45–60	103–128
	Female	208	51–165	76.5 ± 25.9	192	0.56–18.6	2.1 ± 2.3		110–118
*Galeocerdo cuvier*	Male	4	110–200	163 ± 43.8	2	5.34–15.9	10.6 ± 7.5	50–76	300–305
	Female	3	96–168	138.7 ± 37.8	2	2.75–26.9	14.8 ± 7.1		250–350
*Loxodon macrorhinus*	Male	55	41–89.5	64.6 ± 15.0	51	0.22–2.15	0.9 ± 0.6	40–55	62–83
	Female	60	38–92	64.2 ± 16.8	55	0.12–2.78	1.0 ± 0.8		79–90
*Rhizoprionodon acutus*	Male	80	31.3–200	63.0 ± 33.5	77	0.11–19	1.3 ± 2.3	29–40	68–72
	Female	93	31.1–144	59.5 ± 21.5	93	0.11–12.78	1.3 ± 1.7		70–81
*Scoliodon macrorhynchos*	Male	17	26.8–55.6	37.7 ± 9.2	17	0.06–0.71	0.3 ± 0.2	12–15	24–36
	Male	22	28.5–61.9	39.2 ± 6.9	22	0.06–0.97	0.3 ± 0.2		33–35
*Triaenodon obesus*	Male	0						52–60	104–105
	Female	1	120		1	8.62			105–109
*Sphyrna lewini*	Female	73	47–245.3	68.8 ± 26.6	63	0.43–25	1.8 ± 3.1	40–55	140–180
	Male	60	47.1–123	68.0 ± 18.3	49	0.44–3.19	1.3 ± 0.8		200–230

## Discussion

The spatial and temporal variations in diversity and occurrence of sharks were examined in SSCS for four years. Known habitats of the 28-shark species ranged widely from coral reefs, seagrass beds, and pelagic, oceanic and demersal habitats with various depths from epipelagic to mesopelagic zones (Table 1). The sharks were caught from various fishing grounds. In Malaysian waters, at least 63 freshwater and marine water shark species have been reported [20], thus ranking this area fourth in the Southeast Asian region, after Indonesia (111 species), the Philippines (94 species), and Thailand (64 species) [26]. In the present study, Carcharhinidae was the most diverse group with 14 species, followed by Hemiscylliidae (5 species) (Table 2). Of the 28 species found, *Chiloscyllium hasseltii*, *C*. *plagiosum*, *C*. *punctatum*, *Carcharhinus sorrah*, *Loxodon macrorhinus* and *Sphyrna lewini* occurred frequently in the SSCS (Table 2), although the dominant species differed among six landing ports (Fig 2). This occurrence pattern was consistent with a previous study in the same region [16].

Higher diversity was found in the eastern part of the SSCS (East Malaysia) even though we conducted much more intensive surveys and more landing sites in the western part of the SSCS (West Malaysia), which can be explained by the differences in shelf topography and habitat diversity between the western and eastern parts of the SSCS. The broad and shallow Sunda Shelf lies off the east coast of the Peninsular Malaysia (West Malaysia), and the depth in the fishing grounds is shallower than 50 m [27]. The physical features of the seabed vary from the inshore to the deep sea in the Sabah and Sarawak states (East Malaysia). The continental shelf slopes to 200 m depth, while the continental slope dips from 200 m to 800 m depth in the fishing ground of Sarawak State [25]. A deep-sea trench stretches towards Sabah State with depth ranging from 2000 m to 2500 m [28]. In landing ports of the Sabah and Sarawak states, *Squalus altipinnis*, *Heterodontus zebra*, *Alopias pelagicus*, *Halaelurus buergeri* and *Mustelus widodoi* were landed with known habitats on continental and insular shelves and bottom-dwelling habits (Table 1), while shark habitats in the western coast of the Peninsular Malaysia were mainly on coral reefs, seagrass beds and inshore reefs (Table 1). Furthermore, the fishing grounds in West Malaysia are rather limited (22 km (12 nautical miles)), while fishing grounds are rather wide in the Sabah and Sarawak states, where most are more than 22 km [16]. These results suggest that the greater geographic variations and wider fishing grounds in the eastern SSCS might lead to more shark diversity than that in the western SSCS.

Among 11 sharks observed in Terengganu State, three species—*C*. *punctatum*, *C*. *sorrah* and *C*. *hasseltii*—were the dominant species, constituting 90% of the sharks. The seasonal occurrences of sharks were similar among years, although the dominant species were slightly different in different seasons (see Fig 3). These results suggest that migratory patterns and habitat use of sharks in the western coast of the SSCS might be common among species throughout the year with a few seasonal migratory species in the area. Migration of both adult and juvenile sharks is strongly influenced by temperature [29]. However, there is less seasonal seawater temperature fluctuation in tropical waters such as Terengganu State in the SSCS (26.7°C on average, ranging from 25.9°C to 27.6°C) [30]. Therefore, species composition might be similar temporally and spatially in the area. As small-bodied sharks tend to have productive life-history strategies such as early maturity and annual reproduction, moving widely and using additional habitats may benefit these species by increasing foraging success and promoting fast growth [31, 32]. Long-term use of nearshore regions, however, has been reported in some small-bodied species. For example, adult female *Triakis semifasciata* remained in a California estuary for up to 229 days, which was thought to be due to high productivity, with individuals remaining in the region to forage [33]. Long-term presence and high residency of adult *C*. *sorrah* in Cleveland Bay suggests that this nearshore region provides individuals with sufficient prey resources, and perhaps other benefits such as shelter from larger predators [32]. The nearshore region of Terengganu State in the SSCS might also provide sharks with suitable habitat for reproduction and prey resources and the suitable nursery throughout their lives. No similar work in the SSCS or Southeast Asian region is available to compare with the present findings. Further studies regarding baseline biological information such as breeding behaviour, feeding habit, age of maturity, fecundity, growth, habitat and migration are needed to understand the seasonal occurrence patterns of sharks.

Little else is known about the movement and habitat use patterns of sharks in the SSCS, and in the Indo-Pacific waters other than the information on the species composition and diversity. The two bamboo sharks *C*. *hasseltii* and *C*. *punctatum* are bottom dwellers, living in tidal pools on coral reefs, on muddy banks, and amongst mangroves and seagrasses [25]. The spottail shark *C*. *sorrah* occurs over continental and insular shelves [28], including around coral reefs, and moves relatively short distances of ≤ 50 km in nearshore waters, but some have been reported to move distances **>** 1000 km [34]. The bamboo sharks might be more sedentary compared to the spottail shark. Interestingly, however, the size ranges differed between *C*. *punctatum* and *C*. *hasseltii* and *C*. *sorrah* (Fig 4). All the bamboo shark specimens found throughout the studied years were from juvenile to adult stages with no newborn stage (Table 4). The newborn stage less than 20 cm in the bamboo sharks might not be caught by the current fishing methods and gears in SSCS. However, for the spottail shark, 99% specimens found in June and July consecutively for three years were in the newborn juvenile stage (TL < 60 cm), while there were more adult specimens in other months (Fig 4). These results suggest that *C*. *sorrah* might pup in June and July in the western SSCS, and the spawning and/or nursery ground of *C*. *sorrah* might overlap with the fishing ground in Terengganu State. Therefore, a number of newborn juveniles might be fished especially in June and July; however, adult *C*. *sorrah* might migrate widely in the region, and thus, a few adult *C*. *sorrah* occurred throughout the studied years. Differences in life history and habitat use among species and developmental stages might change the seasonal occurrence and species composition in the region.

Seventeen of the 28 observed shark species were landed at various developmental stages from newborn to mature adults (Figs 5 and 6). These results suggest that these sharks hatched around the fishing grounds just before fishing, and the fishing grounds in the SSCS overlap with their spawning, nursery and feeding grounds. Furthermore, *Sphyrna lewini*, *Carcharhinus plumbeus*, *C*. *brevipinna*, *C*. *tjutjot*, *C*. *limbatus*, *C*. *sealei* and *C*. *sorrah* are listed as Endangered, Vulnerable or Near Threatened species [22] (Table 1). Newborn scalloped hammerhead shark individuals were commonly found in 4 of the 6 landing ports in the SSCS (Fig 2). Thus, the current fishing practices may lead declines in shark abundance to critical levels, which may lead to extinction of some species in the future. Furthermore, a number of newborn shark species were fished, suggesting that the populations of those sharks might also be threatened in the future.

Sharks are not targeted by fishers but are caught together with other commercially important species, such as mackerel, scad, sardine and tuna mainly by means of drift gill nets, bottom trawl nets and hook and line in the SSCS region [16, 28]. However, we found at least 28 shark species caught in the SSCS region and many of these sharks were represented by various developmental stages from newborn juveniles to adults at all landing ports. The observed catch suggests that young-of-the-year and fully mature sharks might be captured frequently in the SSCS area. Sharks are predominantly characterized as long-lived and slow-growing, and they produce few offspring. The International Union for Conservation of Nature and Natural Resources [22] reported that over half of the evaluated pelagic shark species are believed to be under threat from extinction [22]. These characteristics are associated with low productivity, close stock recruitment relationships, and long recovery times in response to overfishing. Thus, an improvement in the species selectiveness of the fishing gears and strong enforcement for shark fishing are needed to protect and conserve sharks.

A number of incidents of shark finning and shark specimens without fins were found in the present study (Fig 7B and 7C), although sharks are not officially considered to be targeted by fisheries in the SSCS region. In the SSCS region, all parts of sharks such as meat, liver and fins are fully utilized as food and the inedible parts such as skin and teeth are used for ornamental products and souvenirs [35]. Information on world shark catches and usages is often inadequate and regionally incomplete, despite questions and arguments for sustainability and protection of shark fisheries globally [18, 36, 37]. Bycatch leads to high incidental shark catches, either from general multispecies fisheries or from large-scale to long-range fisheries that target high-value species [38]. Shark fisheries around the world are typically unmanaged and the details of their operations are often not well known. An increasing number of studies reveal shark populations are collapsing in different parts of the world such as the Atlantic Ocean [37–40], the Gulf of Mexico [41], and the Mediterranean Sea [42]. In Southeast Asia, however, most shark fisheries are scarcely documented although sharks are the most significant bycatch species in the region. A large knowledge gap exists regarding shark population trends within Southeast Asia, which is a region of intense human pressure, particularly through unregulated and unmanaged fisheries for sharks. Elasmobranches are not considered to be a highly-priced fishery product globally. Therefore, information related to their biology, fishery and landings is scarce or non-existent.

We observed that 28 shark species at various developmental stages (newborn, juveniles, to fully mature and almost delivering adults) were fished and landed as bycatch products in the SSCS region. The results suggest that the current disorderly fisheries practice might not be regulated and managed in the region for all shark species. Therefore, further fishing pressure can affect shark stock structure, diversity, and biological parameters and, in the worst of cases, could cause a species to become extinct in the future.

## Materials and methods

We observed the sharks at the landing ports in the southern South China Sea (SSCS) between October 2014 and July 2017 (Fig 1). This study was conducted in six sites (states) in the Malaysian South China Sea areas of the eastern coast of the Peninsular Malaysia (West Malaysia) and the western coast of Borneo Island (East Malaysia) for four years (Fig 1). All study sites face the SSCS in Malaysia territorial waters, covering the four states, of Kelantan (5°52´34” N, 102°27´29” E), Terengganu (5°19´20” N, 103°7´43” E), Pahang (3°47´13” N, 103°19´1” E) and Johor (2°38´29” N, 103°39´41” E) in West Malaysia and two states, Sabah (5°58´58” N, 116°4´19” E) and Sarawak (5°58´58” N, 116°4´19” E) in East Malaysia (Fig 1). Specific permissions were granted from the Fisheries Department of Malaysia for the landing ports survey. Shark specimen collection at landing ports did not require permits in Malaysia. Our protocol was in accordance with the guide for animal experimentation of the Universiti Malaysia Terengganu (UMT) and Universiti Brunei Darussalam (UBD), and fish-handling approvals were granted by the animal experiment committees of UMT and UBD. In six sites, monthly surveys were conducted in Terengganu State for approximately four years between October 2014 and July 2017 to understand the spatial and temporal occurrence and diversity of sharks, and other states were sampled one-to-five times (months) during the four years. Overall, we conducted landing port surveys in four sites for 43 times (months) in West Malaysia and seven times at two sites in East Malaysia. All specimens were donated or purchased in the landing ports and were examined on-site at each landing port or were transported to a laboratory for observation. Sharks from SSCS were captured by commercial trawlers, purse seiners, and hook and line fishing as part of the demersal fishery from the coasts to the edge of Malaysia’s Exclusive Economic Zone (EEZ) (Fig 1).

After the sharks were landed, biological parameters, such as total length (TL), body weight (BW) and sex, were measured and observed. We could measure TL for all specimens with fins in caudal and/or tail parts, while BW could not be measured for 74 specimens because the specimens were too heavy and/or their fins had been cut. Twenty external morphometric and meristic characteristics such as colour patterns, shapes, sizes and positions in each shark were measured and observed, and then species identification for each shark specimen was conducted according to Last et al. [25], Ahmad and Lim [43] and Ahmad et al. [44, 45]. During the four years, however we found a number of sharks without fins in caudal and/or tail parts in the East Malaysia (Sabah and Sarawak states); the specimens could not be identified to species level, and we excluded such specimens from the analysis (Fig 7B and 7C).

In Terengganu State, three species, the brownbanded bamboo shark *Chiloscyllium punctatum*, the spot-tail shark *Carcharhinus sorrah* and the Indonesian bamboo shark *Chiloscyllium hasseltii*, were the most dominant species. Their monthly fluctuation of abundance was examined using One-way ANOVA using SPSS (ver. 20.0; SPSS Inc., Armonk, New York, USA). For each variable, a Shapiro–Wilk test was performed to check the normality, and a Bartlett test to check the homoscedasticity. Data that fulfilled the normality and homoscedasticity assumptions were analysed through One-way ANOVA. The differences in the TL across months or years for each month in each sex were examined through a Kruskal-Wallis test in Terengganu State. Consequently, post hoc Mann-Whitney-*U* tests were employed for comparisons.

## Supporting information

S1 Table Shark species studied in this study. (XLSX)

S2 Table Numbers of size distributions of sharks landed in the southern South China Sea. (XLSX)
